# 0716. Does intravenous iron induce oxydative stress in critically ill patients? A comparison with healthy volunteers

**DOI:** 10.1186/2197-425X-2-S1-P47

**Published:** 2014-09-26

**Authors:** S Lasocki, P Piednoir, C Couffignal, E Rineau, C Schilte, G Dufour, X Duval, F Driss

**Affiliations:** Département d'Anesthésie-Réanimation, CHU de Angers, Angers, France; Departement d'Anesthésie Réanimation, CHU Bichat-Claude Bernard-Paris Diderot, Paris, France; CHU Bichat-Claude Bernard-Paris Diderot, Biostatistiques, Paris, France; CHU Bichat-Claude Bernard-Paris Diderot, CIC, Paris, France; Unniversité Paris Nord Val de Seine, INSERM U, 1149 Paris, France

## Introduction

Anaemia is frequent in critically ill patients. Iron deficiency, secondary to blood losses or prior to admission, is in part responsible for this anaemia. Iron may thus be proposed to critically ill patients (CI). However, iron may promote oxidative stress, which is potentially deleterious. In a mouse model, we previously demonstrated that iron induces less oxidative stress in inflamed mice than in control ones [1], but no data are available in the CI.

## Objective

To compare the oxydative stress induced by an intravenous infusion of iron in CI and healthy volunteers (V).

## Methods

Adult critically ill patients (from 2 ICUs) and healthy Volunteers were included after informed consent. Blood samples were drawn before (T0) and 2, 6 and 24 hours (T2, T6, T24) after an intravenous infusion of 100 mg of iron sucrose (Venofer©) over 60 minutes. Markers of lipid oxidation -8α-Isoprostanes (8ISO)-, protein oxidation -Advanced Oxydized Protein Product (AOPP)- as well as glutathion reduced/oxidized (GSH/GSSG), Non-transferrin bound iron (NTBI) have been measured at these time points. Variations of area under the curves from T0 to T6 (ΔAUC_0-6_) have been compared using a Wilcoxon test. Data are expressed as n(%), mean±SD or median[min-max].

## Results

38 CI have been studied (25(66%) males, aged 67.9[19-85]yrs, 38(100%) ventilated, SAPSII 48.5[21-80], Hb 8.4[6.6-11.8] g/dl) and 39 V (18(46%) males, aged 42.1[21-78] yrs, Hb 13.9[11.9-17.2] g/dl). Iron treatment indications for CI were (many causes possible): 18(45%) elevated soluble transferrin receptor (sTfR), 14(35%) ferritin < 100 µg/l + TSat< 20%, 12(30%) blood loss > one blood mass, 9(22%) elevated sTfR/log Ferritin ratio. At T_0_, [8ISO] was higher in CI than in V 8.48[3.1-63.4] vs. 4.51[2.05-13.33] pmol/l), but the ΔAUC_0-6h_(8ISO) was not different (p=0,38). Only the ΔAUC_0-6h_ (GSH) was lower in V (p=0,009), arguing for a more important decrease in anti-oxidant defences. The table summarized all the results. Eight CI had a second set of dosages (after the 4^th^ iron infusion) showing no difference in any markers compared to the first set of dosages.

**Table 1:** Oxidative stress markers dosages.

	Critically ill (n=38)	Voluntaries (n=39)	P
ΔAUC0-H6 (8ISO)	0.06 [-86.51 - 29.45]	2.01[-16.60 - 17.95]	0.38
C0 AOPP (µg/l)	36.57 [17.46 - 98.90]	19.07 [10.49-34.92]	
ΔAUC0-H6 (AOPP)	14.8 [-172.3 - 110.1]	12.4 [-92.7 - 63.7]	0.69
C0 GSH (µg/l)	3.97 [1.49 -7.31]	1.22 [0.66 -2.12]	
ΔAUC0-H6 (GSH)	-8.34 [-615.4 - 329.36]	-142.4 [-1640.1 - 989.1]	0.009
C0 GSSG (µg/l)	94.90 [31.80 - 349.00]	414.2 [266.3 -845.5]	
ΔAUC0-H6 (GSSG)	1.44 [-163.17 -77.26]	-11.79 [-152.85 -116.36]	0.43
C0 NTBI (µg/L)	0.23 [-1.44 - 0.75]	.22 [-1.35 -0.89]	
ΔAUC0-H6 (NTBI)	0.74 [-3.75 - 9.53]	0.33 [-4.53 -9.77]	0.35

## Discussion

We haven't seen any increase in lipid (8ISO) or protein (AOPP) oxidation in CI compared to V. On the contrary, V had a greater decrease in anti-oxidant (ie GSH), suggesting higher oxidative stress. Iron in the CI doesn't induce more oxidative stress than in V, but other studies are needed to confirm the absence of clinical toxicity.
